# Organism-Level Tumor Models in Zebrafish Danio rerio

**Published:** 2018

**Authors:** I. V. Mizgirev, D. R. Safina, I. V. Demidyuk, S. V. Kostrov

**Affiliations:** N.N. Petrov National Medical Research Center of Oncology, Ministry of Health of Russia, Leningradskaya Str., 68, Pesochnyy Settlement, St. Petersburg, 197758, Russia; Institute of Molecular Genetics, Russian Academy of Sciences, Kurchatov Sq., 2, Moscow, 123182 , Russia

**Keywords:** Zebrafish, Danio rerio, molecular oncology, syngeneic, allogeneic and xenogeneic transplantation, transparent fish lines, bioimaging

## Abstract

Development and implementation of adequate organism-level models is one of the key elements in biomedical research that focuses on experimental oncology. Over the last decade, studies using Zebrafish (*Danio rerio*) have gained in popularity in this area of research. This review describes the various approaches that have been used in developing highly effective models for oncological (clinical term, better cancer or tumor) studies based on *D. rerio*. Priority is given to transplantation models of cancer and their application to optically transparent *D. rerio *lines, including clonal ones, and utilization tumors of various origins bearing fluorescent labels. The combination of tumor transplantation at organism-level models in transparent clonal *D. rerio *lines with fluorescent microscopy, FACS-fractionation of tumor cell subsets, and transcription analysis can result in one of the most promising research approaches in providing new information on tumor formation and growth.

## INTRODUCTION

Cancer is one of the most significant problems of modern medicine. Cancer mortality ranks second in industrial countries and is projected to claim first rank in the future [1]. In this context, the development of biological models providing unique opportunities for studying the mechanisms of initiation and progression of malignant neoplasms in order to improve the effectiveness of novel antitumor therapies is among the top priorities in modern oncology.

Most of the current approaches are based primarily on the use of human and other mammalian cells *in vitro*. Despite their obvious advantages, these models have a number of significant limitations. First of all, the results obtained using these approaches are out of the whole organism context. For example, 1) *in vitro *models do not reflect the developmental stage or age of the organism; and 2) it is impossible to evaluate *in vitro *some organism-mediated effects on tumor growth, such as the influence of the tissue microenvironment, the hormonal and metabolic status, the immune system, etc. Obviously, *in vitro *studies should be supplemented with *in vivo *organism-level models. Rodents are the central organism-level model in cancer research. Transplantable tumor lines that can be serially engrafted to inbred recipients are considered as the “gold standard” in experimental oncology, since they provide high penetrance of synchronously developing tumors. On the other hand, standard *in vivo *tumor growth models using rodents are hard to adapt to modern technologies for high-throughput screening of antitumor agents because of the high cost and labor-intensity of such studies. It should also be emphasized that the model does not allow high-throughput bioimaging of tumor development, including the changes taking place in the tumor microenvironment *in vivo*, which is a significant drawback in the analysis of fine mechanisms of tumor progression. Therefore, researchers have begun focusing on alternative tumor models which can compete with rodents in terms of the translational significance of experiments for clinical practice and that are much more informative and efficient.

In recent years, the freshwater fish *Danio rerio *(zebrafish) has become an increasingly popular model. This is due to its small size (2.5–4 cm), relatively short lifespan, as well as the possibility of laying up to several hundreds eggs per week from one female, *ex utero *development, transparency of embryos and larvae, the relative simplicity of maintenance and breeding, and the existence of numerous mutant and transgenic lines.

It should be emphasized that the *D. rerio *model is perfectly adapted to the use of modern molecular and genetic. The genome of this organism has been mapped and sufficiently annotated [www.ncbi.nlm.nih.gov/genom/GRCz11]. Methods for precise editing of the zebrafish genome have become relatively simple and efficient, including targeted mutations using the ZFNs (zinc finger nucleases) platform [2-4] and the recently thoroughly elaborated CRISPR/Cas9 system [8]. The method of high-throughput insertional mutagenesis using retroviruses [5] and transposon elements has also been developed [6, 7]. Various genetically engineered *D. rerio *lines harboring oncogenes have been developed to induce tumors. Several of these tumors have been adapted to transplantable models. Recently, considerable attention has been focused on xenograft transplantation of human tumor cells.

## D. rerio MODELS BASED ON INDUCED TUMORS

**Fig. 1:**
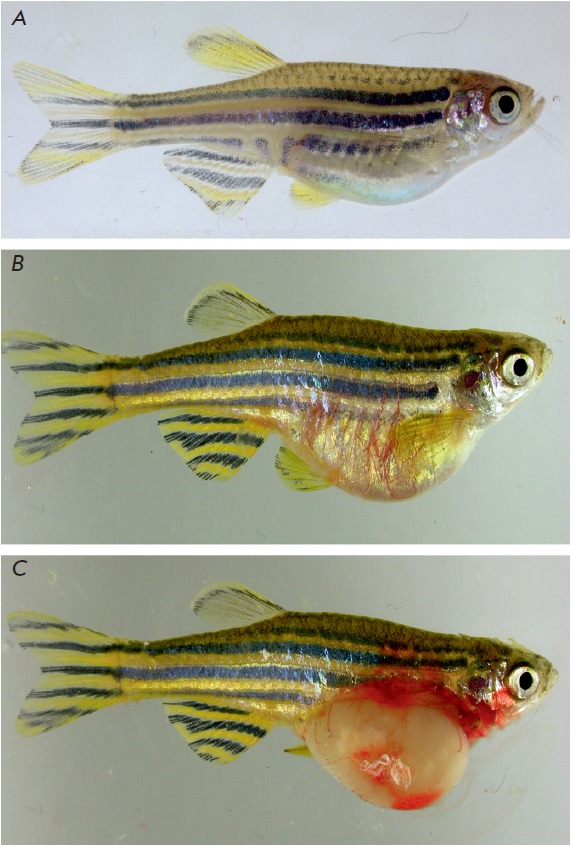
*D. rerio *with a carcinogen-induced hepatic tumor. A – healthy fish; B – fish with induced hepatocarcinoma; C – the same fish with an opened abdominal cavity. Tumor induction was carried out according to the procedure described in Khudoley [10].

The pioneering studies by Stanton [9] and Khudoley [10] on hepatic tumor induction with chemical carcinogens laid the foundation for tumor growth modeling in *D. rerio*. This model remains one of the most popular tools used to study various aspects of tumor growth in fish (*Fig. 1*).

Furthermore, many new models have been generated by introducing vector DNA containing different oncogenes controlled by tissue-specific promoters into *D. rerio *zygotes [11], and they resulted in efficient induction of embryonic rhabdomyosarcomas [12], melanomas [13], hepatocellular carcinomas [14], and various types of leukemia [15-17].

Importantly, many genetically induced tumors express fluorescent protein-reporters, which enable to determine the time of tumor onset and investigate its growth and dissemination based on increased level and spatial distribution of fluorescence [18]. Some of these models use genetic constructs with regulatory elements to control the timing of tumor onset. In particular, induction of hepatocellular carcinomas by KRASV12 under the control of doxycycline [19] and mifepristone [20] has been developed. However, most of the above-mentioned models still possess limitations, such as a low incidence and long and variable latent period of tumor onset, which makes it difficult to use them, for example, for the screening of potential drugs.

## TRANSPLANTATION MODEL BASED ON D. rerio

Studies aimed at developing malignant growth models based on the transplantation of labeled mammalian or fish tumor cells into the organism of *D. rerio *are being carried out in many laboratories around the world [21-25].

However, until recently, all attempts at using transplantation models based on *D. rerio *in cancer research faced a number of significant limitations. In particular, for a long time, a tumors could only be transplanted to sublethally irradiated fish or embryos at the early developmental stages.

At the same time, the approach associated with the use of sublethal gamma irradiation was not that convenient due to the high mortality of fish, as well as the quite rapid recovery of the immune system in survived animals [26].

The technique of xenogeneic transplantation of tumor cells, including human [27] and rodent [28] cells, into *D. rerio* embryos seems more attractive and has witnessed intensive development in recent years. Tumor cells transplanted into embryos at early developmental stages (before the age of 48 hours) are not rejected because of the immaturity of the embryonic immune system and can survive in the recipient organism for several days. In some cases, these cells may migrate (“metastasize”) some distance from the injection site [29] and produce angiogenic factors stimulating the growth of blood vessels [30-32]. Nevertheless, this type of transplantation models also exhibit some limitations related, for example, the temperature conditions optimal for fish embryos at 28°C, which are not optimal for mammalian cell growth. However, it should be noted that *D. rerio *can be maintained for some time at a higher temperature, up to 35°C, which is more adequate for mammalian cells, without significant loss in their survival rate [33]. Recently, xenotransplantation of tumors into *D. rerio* embryos has been successfully used to assess the sensitivity of tumors obtained from patients to the action of various drugs and their combinations in order to select the optimal strategy of drug therapy [34]. Previously, such studies were carried out only in athymic NOD/SCID mice. However, the prospects for a widespread use of this approach in clinical oncology are extremely limited due to its high cost and the labor-intensity of such studies.

Unfortunately, all attempts at developing inbred *D. rerio *lines similar to inbred mammalian ones using standard inbreeding techniques have failed due to the reduced fertility of fish after several rounds of closely related crossing.

The problem of heterologous tissue transplantation into the organism of *D. rerio *has largely been solved thanks to the development of three new experimental approaches. The first one is based on the production of homozygous diploid clonal lines of *D. rerio* [35], which for the first time enabled the transplantation of tumor or normal cells from one fish to another within the same line without graft rejection. The possibility of constructing such lines was first demonstrated by Streisinger et al. [36]. The double-heat shock method was used to generate clonal lines [37]. For this purpose, oocytes from *D. rerio *were fertilized *in vitro *with UV-inactivated sperms and then subjected to a short thermal shock to block the first cell cleavage. Survived embryos (approximately 0.5% of heat-shock treated zygotes) were grown to adult state. This procedure leads to the development of completely homozygous diploid fish, which, nevertheless, are genetically different from each other. At the second stage, the oocytes obtained from each of the homozygous females are subjected to the next round of fertilization with UV-irradiated sperm, followed by heat shock. The offspring obtained from each homozygous female are genetically identical (clones) to this female and to each other because of the initial homozygosity of the mother organism. Clonal lines are further maintained by crossing the fish within one clone with each other. It should be noted that the sex of *D. rerio *is determined not by sex chromosomes but by physiological factors acting at early developmental stages, and, therefore, the offspring produced by clonal fish crossing will include both males and females. These lines are characterized by complete genetic identity and full homozygosity of the animals within each clone. This is a direct analogue of inbred rodent strains.

Clonal zebrafish lines consisting of genetically identical animals have been proven to be a convenient model for serial transplantation of tumor cells. Some tumor strains originating from the nitrosodiethylamine-induced hepatic and pancreatic carcinomas of clonal fish have undergone more than 20 consecutive passages without signs of rejection. In later studies, the clonal fish lines CG1 and CG2 were used to induce and subsequently transplant fluorescent reporter-labeled rhabdomyosarcoma [38] and leukemia cells [39] to syngeneic recipients. In that situation, the small sizes of the *D. rerio *larvae and embryos make them an ideal tool for large-scale transplantation of tumor cells to hundreds of syngeneic recipients within a short period of time.

The second approach, which has been under development since recently, is based on the use of immunodeficient fish lines [40] similar to athymic NOD/SCID mice [41]. The model enables quite effective allogeneic transplantation of malignant and normal tissues to a recipient. However, it must be emphasized that immunodeficient animals cannot be used to study, for example, a number of the aspects of tumor interaction with the host organism.

In this regard, another approach based on the recently developed double transplantation technique appears especially promising [42]. The approach is based on engraftment of lethally irradiated tumor cells into *D. rerio *embryos at the early developmental stages (up to 48 hours after fertilization). It has been shown that these cells persist in the recipient organism for about 2 weeks, do not affect its viability but lead to the development of specific immunological tolerance to this tumor without causing global immunodeficiency. Three months after primary transplantation, these animals can be injected with non-irradiated cells of the corresponding tumor. These cells successfully form tumor nodes and are capable of metastasizing. The approach was tested on various human tumor cell lines, including hepatocarcinoma and prostate cancer. Thus, this model enables transplantation of allogeneic and xenogeneic tumors to adult fish and quite accurately simulates spontaneous tumor growth in the host organism.

## TRANSPLANTATION MODELS BASED ON TRANSPARENT LINES OF D. rerio

**Fig. 2:**
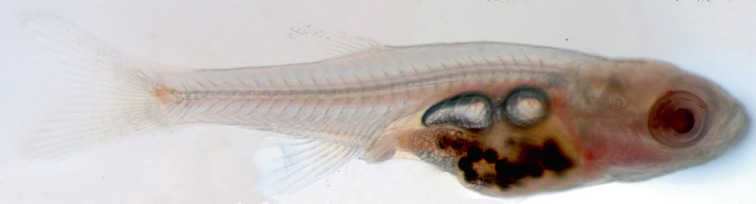
Three-week old *D. rerio, *transparent line *sheer*.

**Fig. 3:**
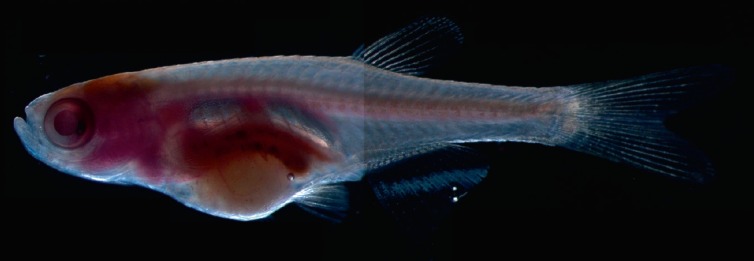
Carcinogen-induced hepatocellular carcinoma in a transparent *sheer *line of *D. rerio*. Tumor was induced according to the procedure described in Khudoley [10].

The loss of fish body wall transparency with growth due to the production of pigment cells, i.e. chromophores producing black (melanophores), light-reflecting (iridophores), and yellow (xanthophores) pigments in the skin, eyes, and peritoneal lining, is another limitation in the use of *D. rerio *as an organism-level tumor model. This significantly complicates the bioimaging analysis of the development of the transplanted or induced tumors in the animal’s body. However, optically transparent lines (*ruby*, *casper*, *sheer*) have been developed [43, 44] which lack most pigment cells and, as a result, have transparent body walls through which all visceral organs, as well as transplanted normal and tumorous tissue, can be visualized (*Fig. 2*, *Fig. 3*). Therefore, transparent lines are an almost ideal model for real-time non-invasive study of tumor growth *in vivo*. Obviously, generation of clonal optically transparent lines, as well as combining transparent lines with double-transplantation technology, should be the next step.

**Fig. 4:**
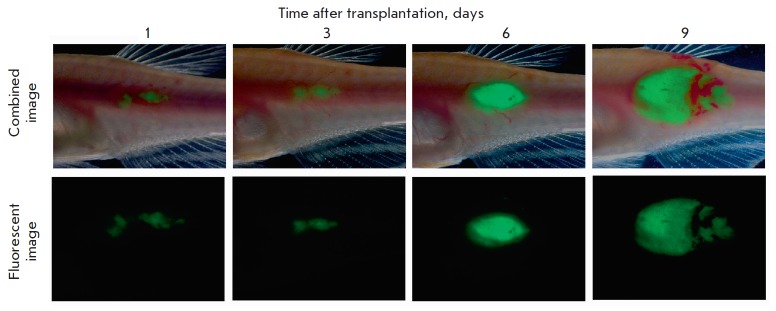
Green fluorescent protein-labeled rabdomiosarcoma grown in the clonal line of *D. rerio*. Intramuscular transplantation, the 4^th^ passage.

Generation of optically transparent clonal *D. rerio *lines makes the use of fluorescently labeled tumors for transplantation highly promising. Generation of these tumors by mosaic expression of transgenes containing various oncogenes, in combination with fluorescent reporters, has been demonstrated previously for clonal *D. rerio *lines [38, 39]. Chemical carcinogen-induced tumors in fish which are phenotypically very similar to human tumors might be very desirable for bioimaging of cancer development and progression. This is being accomplished by chemical carcinogenesis in transgenic *D. rerio *sublines based on clonal line CG2 expressing a fluorescent marker ubiquitously: i.e, in all tissue types. Any tumor induced in fish that belong to one of these sublines will bear a fluorescent label and can be transplanted to non-trasgenic syngeneic CG2 fish (*Fig. 4*). The implementation of this technique in clonal transparent lines of *D. rerio *will result in a transplantation model that provides exceptional capabilities for detailed bioimaging of tumor growth.

## PROSPECTS OF MODEL DEVELOPMENT

One of the most interesting strategies in the development of models based on *D. rerio *includes its combination with modern approaches to transcriptome analysis. At present, transcriptome analysis of a number of tumors of *D. rerio *of various geneses (hepatocellular carcinoma, melanoma, rhabdomyosarcoma, etc.) is carried out using microarrays and RNA sequencing technology. The results have been compared to those obtained when analyzing corresponding human tumors. The finding that the transcriptome changes accompanying tumor growth in humans and *D. rerio *are conserved was the main conclusion of these studies [45, 46]. This conclusion is extremely important for further development of this system, since it indicates the possibility of its use for a detailed analysis of the mechanisms of the onset and development of human tumors and high-throughput screening of antitumor agents. Detailed study of the interaction between the tumor and surrounding stromal tissue is one of the most promising areas, which provides hope for the development of new approaches to the therapy of tumor diseases [47]. To date, it is clear that a microenvironment represented primarily by fibroblasts, endothelial cells, pericytes, leukocytes, and the extracellular matrix is an integral part of a tumor and is directly involved in the control over its formation, growth, and progression. In turn, tumor cells have an active remodeling effect on the surrounding tissue. Therefore, the tumor growth process involves a complex set of various interactions that change with tumor progression. Obviously, any analysis of the interaction between a tumor and stromal tissue is impossible without the use of organism-level models. The most common, currently used systems are based on immunodeficient rodent lines, which provide a combination of an organism-level model, fluorescent microscopy, FACS analysis of cell subpopulations, and transcription analysis [48]. However, the *D. rerio *model is gaining in popularity, especially where high-resolution microscopy is warranted [49, 50]. It is very likely that implementation of these approaches, in combination with a syngeneic transplantation model based on optically transparent *D. rerio *lines, will provide fundamentally new insight into understanding tumor growth and its interaction with its microenvironment.
